# Galvanism in Uterine Hemorrhage

**Published:** 1845-06

**Authors:** 


*Galvanism in Uterine Hemorrhage.* (*Prov. Med. and Surg. Journ.*)—Dr. Thomas Radford has employed galvanism with great success in the treatment of cases of uterine hemorrhage, accidental or unavoidable, accompanied by exhaustion, and occurring before, during or after labour. "I am satisfied," he says, from positive trial of the remedy, that it will be found a most important agent in tedious labour, depending upon want of power in the uterus, and where no mechanical obstacle exists. I would also suggest the probability of its proving valuable in originating uterine action *de novo*, in cases where it may be considered necessaay to induce premature labour. It seems to me also to be worthy of trial in certain cases of monorrhagia in the ungravid state, where, on vaginal examination, the uterus is found to be atonic, as evidenced by its large flaccid condition, and the patulous state of the os uteri.

His mode of applying galvanism is the following:—"The brass ball of the vaginal conductor is to be passed up to the os uteri, and moved about, at intervals, on to various parts of this organ; at the same time, the other conductor must be applied to the abdominal parietes over the fundus uteri. Shocks may be also passed transversely through the uterus, by simultaneously applying the conductor on each side of the belly.

The application should be used at intervals, so as to approximate in its effects, as nearly as possible, to the natural pains. It may be continued until it meets the exigencies of the case.—*Ibid.*

